# Progressive alteration of murine bladder elasticity in actinic cystitis detected by Brillouin microscopy

**DOI:** 10.1038/s41598-023-51006-2

**Published:** 2024-01-04

**Authors:** Laura Martinez-Vidal, Claudia Testi, Emanuele Pontecorvo, Filippo Pederzoli, Elisa Alchera, Irene Locatelli, Chiara Venegoni, Antonello Spinelli, Roberta Lucianò, Andrea Salonia, Alessandro Podestà, Giancarlo Ruocco, Massimo Alfano

**Affiliations:** 1 Division of Experimental Oncology/Unit of Urology, IRCCS Ospedale San Raffaele, 20132 Milan, Italy; 2 Università Vita-Salute San Raffaele, Via Olgettina, 60, 20132 Milan, Italy; 3 Center for Life Nano- and Neuro-Science, Istituto Italiano di Tecnologia, Viale Regina Elena 291, 00161 Roma, Italy; 4 CrestOptics S.p.A., Via Di Torre Rossa, 66, 00165 Roma, Italy; 5 Experimental Imaging Centre, San Raffaele Scientific Institute, Via Olgettina 60, 20132 Milan, Italy; 6 Pathology Unit, IRCCS Ospedale San Raffaele, 20132 Milan, Italy; 7 Dipartimento Di Fisica “Aldo Pontremoli” and CIMAINA, Università Degli Studi Di Milano, 20133 Milan, Italy; 8 Dipartimento Di Fisica, Universitá Di Roma “La Sapienza”, Piazzale Aldo Moro, 5, 00185 Roma, Italy

**Keywords:** Biological physics, Techniques and instrumentation, Bladder, Imaging, Microscopy

## Abstract

Bladder mechanical properties are critical for organ function and tissue homeostasis. Therefore, alterations of tissue mechanics are linked to disease onset and progression. This study aims to characterize the tissue elasticity of the murine bladder wall considering its different anatomical components, both in healthy conditions and in actinic cystitis, a state characterized by tissue fibrosis. Here, we exploit Brillouin microscopy, an emerging technique in the mechanobiology field that allows mapping tissue mechanics at the microscale, in non-contact mode and free of labeling. We show that Brillouin imaging of bladder tissues is able to recognize the different anatomical components of the bladder wall, confirmed by histopathological analysis, showing different tissue mechanical properties of the physiological bladder, as well as a significant alteration in the presence of tissue fibrosis. Our results point out the potential use of Brillouin imaging on clinically relevant samples as a complementary technique to histopathological analysis, deciphering complex mechanical alteration of each tissue layer of an organ that strongly relies on mechanical properties to perform its function.

## Introduction

The biological consequences of altered tissue mechanics greatly affect organ homeostasis and function^1^. In vivo, the local microenvironment continuously exerts mechanical forces (i.e., shear, compressive, extensional forces) on cells, triggering the activation of a cascade of biomechanical pathways critical for tissue morphogenesis, homeostasis and development^2^.

**Figure 1:**
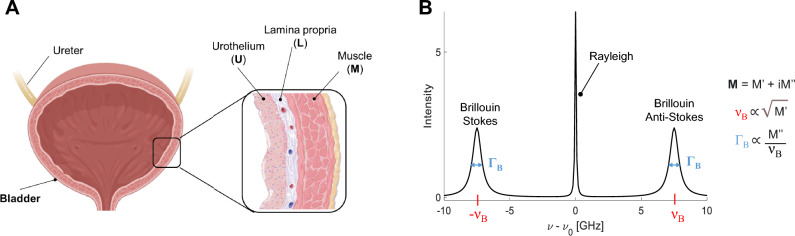
Bladder anatomy and basic Brillouin theory to measure bladder mechanics. (**A**) Schematic representation of the bladder wall: the inner layer of the bladder is the urothelium (U), a cell layer formed by epithelial cells, which sits on the basal membrane; the lamina propria (L) is mainly formed by extracellular matrix (ECM); below the lamina propria there are several concentrical layers of muscle tissue (M), which allow for bladder contraction and urine storage and expulsion. Image taken from BioRender. **B)** An example of Brillouin spectrum. ν_B_ = Brillouin shift; Γ_B_ = Brillouin full width at half maximum; M = complex Longitudinal Modulus, having a real (M’) and imaginary (M’’) part. A Brillouin spectrum consists of a central Rayleigh peak, due to photons scattered at the same frequency of the incident radiation ν_0_, and two Brillouin peaks, due to photons scattered at lower (Stokes) and higher (Anti-stokes) frequencies than the incident one. The Brillouin peaks are identical, located in the GHz range, centered at ±|ν_B_| and have a full width at half maximum equal to Γ_B_.

The bladder is an excellent example of an organ in which mechanical properties regulation is essential, as it must adapt to the internal urinary volume with elastic extension or compression. The bladder is a multilayered organ (Fig. 1A): the most internal layer is the urothelium (U), constituted by epithelial cells^3^; the basement membrane separates the urothelium from the lamina propria (L), which is mainly composed of extracellular matrix (ECM), including collagen I and III, elastic fibers, blood vessels, unmyelinated nervous endings and some discontinuous muscle bundles^4,5^; the external part is formed by concentric layers of smooth muscle (M), surrounded by an outermost layer of perivesical adipose tissue^4^.

Actinic cystitis is one of the most common complications of radiation therapy^6^. This pathological condition affecting the bladder and concerning its mechanical properties alteration is a side effect of pelvic X-ray radiotherapy, commonly used to treat prostate, colorectal, cervical, and ovarian cancer^7,8^. Actinic cystitis is caused by the accumulation of ECM proteins due to chronic inflammation^9^. Its prognosis includes first a short acute phase, in which X-ray radiation causes cell membrane and DNA damage, as well as water radiolysis resulting in free radical formation^10^. Therefore, the loss of umbrella cells covering the urothelium exposes the urothelial cell layer to urine, which further activates inflammation mechanisms, resulting in a chronic inflammatory stage^11^. Finally, due to lack of oxygen supply after ischemia of microscopical blood vessels, the tissue becomes atrophic, with increased deposition of collagen within the lamina propria and between muscle bundles^10^, increased fibroblast and myofibroblast infiltration, decreased cellular muscle relaxation, development of tissue edema and presence of massive fibrosis^7^. Actinic cystitis ultimately results in organ failure, with an important impact on the quality and amount of life of the patient^1^, which in some cases may lead to radical cystectomy^12^.

The characteristic accumulation of collagen and the resultant fibrotic tissue remodeling alter the mechanical properties of the tissues, ultimately resulting in bladder function impairment^6^. Understanding the progression of such mechanical alteration is crucial, as well as assessing whether mechanical alteration could be used as a prognostic marker or indicator of tissue priming for disease development, eventually setting the path for early diagnostic/prognostic tools.

Previous studies have shown that scarring and thickening of bladder tissue leads to decreased bladder contractility, resulting in function impairment^9,10,13^. Nevertheless, systematic studies characterizing how bladder mechanical properties are altered at the microscale and considering the kinetics of the disease are still missing. In this context, we recently showed that Young’s Modulus, which describes tissue rigidity, increases in X-ray irradiated murine bladder^14^. This study describes bladder tissue elasticity at the microscale, dissecting the contribution of the different bladder layers to the mechanics alteration.

It has been previously reported that different collagen amounts affect both tissue stiffness and viscosity in cellular aggregates^15^ and that, in general, cells and tissues are highly sensitive to both elastic^16,17^ and viscous^18^ properties of the environment. As Young’s Modulus only provides partial insight into the static mechanical properties of a material, it is crucial to characterize layer-specific mechanical alterations of the bladder further, considering its viscoelastic tissue properties.

This study aims to detect the changes in the viscoelastic properties in bladder tissues by exploiting Brillouin Microscopy, a cutting-edge imaging technique that provides a complete characterization of the viscoelastic properties of a sample through the analysis of its Brillouin spectra. In viscoelastic theory, the mechanical response of a material to an external perturbation is modeled as a combination of two ideal characteristics, those of an elastic solid and a viscous liquid^2^. From the Brillouin spectrum (Fig. 1B) it is possible to quantify the complex Longitudinal Modulus **M**, composed of a real part (*M’*), representing the elastic response, and an imaginary part (*M’’*), representing the dissipative response of the material. This spectrum arises from the scattering of photons upon interaction with the sample’s longitudinal acoustic phonons: it consists of a central Rayleigh peak, due to photons scattered at the same frequency of the incident radiation ν_0_, and two Brillouin peaks, due to photons scattered at lower (Stokes) and higher (Anti-stokes) frequencies than the incident one. These peaks are identical, located in the GHz range, centered around the Brillouin frequency shift (ν_B_, directly dependent on M’) and characterized by an equal full width at half maximum (Γ_B_, directly dependent on M’’).

Applications of Brillouin microscopy are emerging in the mechanobiology field to assess biomechanical parameters of different cells ^19–25^ and tissues^26–32^ under physiological and pathological conditions, highlighting its potential diagnostic use. Indeed, it is a label-free technique, as it is based on a spontaneous process. Similarly to confocal laser-scanning microscopy, it uses a laser beam as a probe: it is thus contactless, non-invasive, and it allows for optical sectioning of focal planes. By scanning the sample, it is possible to map its Brillouin spectra point-by-point, in three dimensions and with a confocal approach^33^, i.e., with sub-micrometric spatial resolution. In such a manner, Brillouin Microscopy can resolve tissue mechanics at the microscale, a quality still lacking in many modern measuring methods for biomechanics. In the bladder, this is a fundamental characteristic that may assess the contribution of the different anatomical components to the overall mechanical alteration in disease.

Here, we established associations between bladder X-ray irradiation leading to fibrosis and bladder tissue mechanics by exploiting our custom-built Brillouin Microscope. We first investigated the physiological effects of aging on *ex-vivo* rat bladder mechanical properties, finding slight differences in the Brillouin shifts of lamina propria in rats at different ages. We then investigated murine bladders in X-ray-induced cystitis at different time points post-irradiation. We report a significant correlation between Brillouin shift and fibrosis condition, showing lower Brillouin shifts in the lamina propria of fibrotic bladders. Finally, we compared the Brillouin dataset to already published data, performed on the same bladder, through atomic force microscopy (AFM) and AFM-derived nanoindentation techniques^14^, which remain the gold-standard technique to characterize the mechanical properties of cells and tissues^34^, and we discussed the differences between the two techniques. In such a manner, we assessed the potential use of Brillouin Microscopy as a diagnostic tool for biomedical imaging purposes by a label-free and quantitative characterization of mechanical changes of bladder tissues upon radiation exposure.

## Results

### Brillouin imaging of the healthy bladder wall shows differences in mechanical properties of the different tissue layers

**Figure 2:**
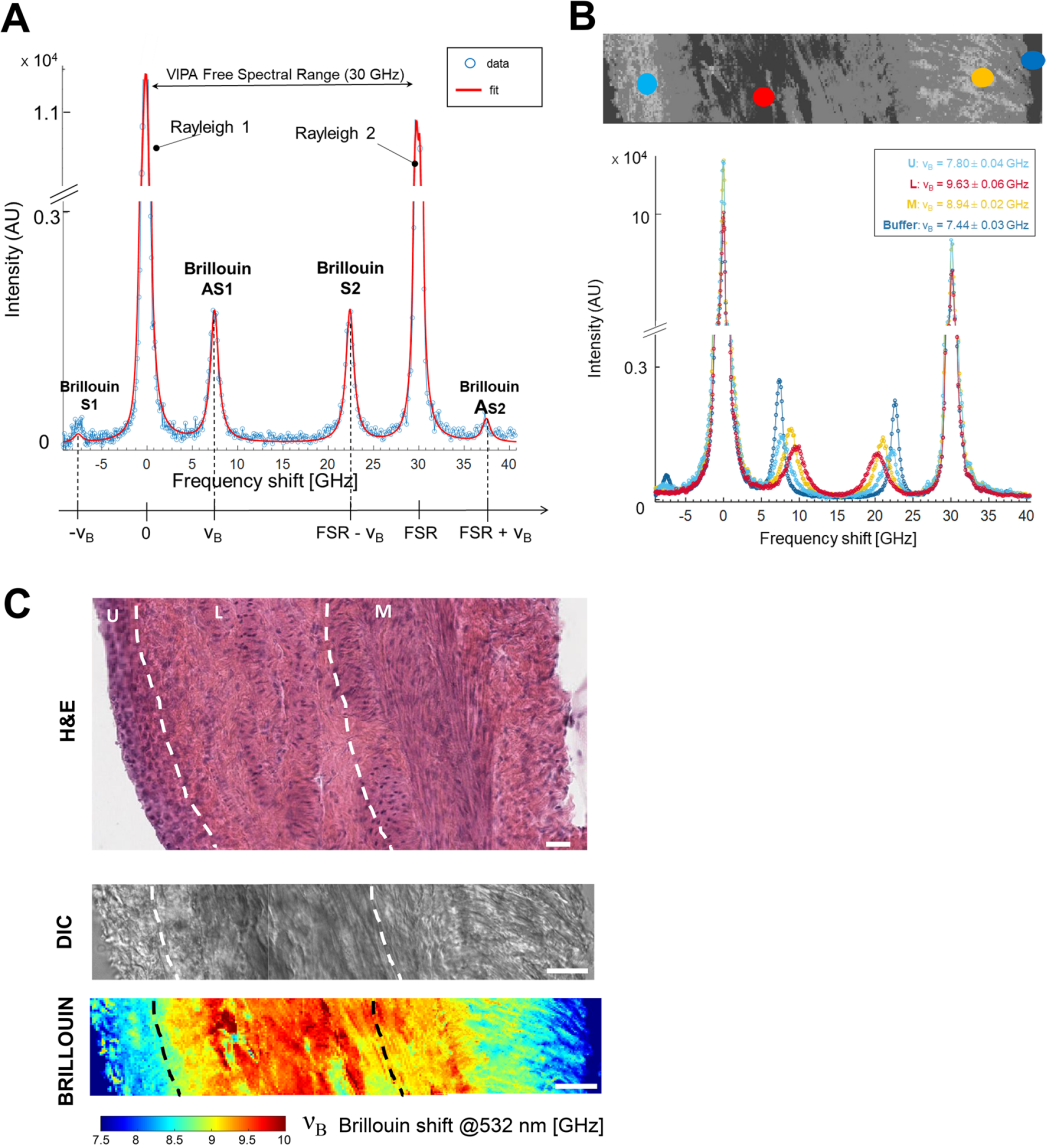
Brillouin imaging of the healthy bladder wall. (**A)** Typical Brillouin spectrum of water obtained with our Brillouin microscope. The spectrum is composed by 2 Rayleigh peaks of adjacent dispersion orders (fixed at 0 and FSR = 30 GHz), and 4 Brillouin peaks whose location is clarified underneath the x-axis. In backscattering configuration and λ = 532 nm, water *ν*_*B*_ = 7.40 GHz. (**B**) DIC image of murine bladder wall. Example of 4 Brillouin spectra obtained from different regions of a bladder section. U: urothelium, L: lamina propria, M: muscle, buffer is PBS. (**C**) Bladder wall imaging by different microscope techniques. Scale bars = 20 µm. *Upper panel:* HE staining of the healthy rat bladder wall, showing the different anatomical bladder tissue layers, as in Fig. 1. *Lower panel:* DIC image and corresponding Brillouin maps of healthy bladder walls from urothelium (left) to muscle (right). FSR: Free Spectral Range. DIC: differential interference contrast microscopy. HE: hematoxylin eosin staining. AU: arbitrary unit.

Our custom-built Brillouin Microscope (Figure S1A, Methods section) consisted of an inverted standard microscope coupled to a single virtually imaged phased array (VIPA)-based spectrometer^35^. Confocal sectioning of the sample was ensured by single-mode fibers, providing (with a laser wavelength of 532 nm and a NA = 1.40 objective) an axial resolution of the maps of around 500 nm^36^ *.*Through the transmission of the VIPA interferometer, the Brillouin triplet (shown in Fig. 1B) was repeated every 30 GHz; thus, our experimental Brillouin spectra consisted of 2 Rayleigh peaks of adjacent dispersion orders and a total of 4 Brillouin peaks (Fig. 2A,B), with a spectral precision of 16 MHz (Figure S1B) and a signal to noise ratio (SNR) equal to 17 (Methods section). Our Brillouin Microscope also featured a standard widefield transmission unit for images in Differential Interference Contrast (DIC) mode, which provided a morphological insights of the sample.

50 µm thick bladder sections, attached to polarized glass slides, were measured after thawing and OCT removal with PBS buffer, then were left in PBS during acquisitions. We measured all the samples at a depth of 5 microns inside the sample (see Methods).

With our Brillouin microscope, we recovered *ν*_*B*_ maps for every sample under investigation. This enabled the estimation of mechanical features of murine bladder walls in a label-free and contact-less fashion, while tissues were kept fresh and hydrated during acquisitions. These maps covered the whole width of the bladder wall and were collected with 800 nm longitudinal step size: this enabled a sub-micrometric spatial resolution in all three dimensions, much more accurate than the macroscopical ones available with other techniques of mechanobiology^13,37^. In such a manner, our spatial resolution was high enough to separate the mechanical contribution of each layer across the bladder. In the rare cases in which sample spatial changes were on a length scale lower than our resolution, we observed the formation of blended spectra (like the one shown in Figure S2)^38^. Indeed, different regions of the bladder provided different Brillouin spectra, whose shape changed significantly both in *ν*_*B*_ and in Γ_B_, closely related to the local biophysical properties (Fig. 2B). Indeed, raw Brillouin spectra of different tissue layers of the specimen highlighted the sensitivity of the technique to their various biophysical components with high signal-to-noise ratio (Fig. 2B, cyan red and yellow dots), and the signal coming from PBS buffer (Fig. 2B, blue dot) confirmed that the tissue was kept hydrated during the whole acquisition.

We found that the bladder wall is a mechanically inhomogeneous material whose mechanical properties are well correlated with different tissue layers (Fig. 2C), as confirmed by Hematoxylin and Eosin (H&E) staining, performed on the same tissue after Brillouin acquisitions.

DIC images, acquired simultaneously to Brillouin data, showed very little contrast and poor sensitivity to sample morphology (Fig. 2C).

The urothelium exhibited the lowest Brillouin shift values, ranging from 7.5 to 8.5 GHz (Fig. 2C), which are typical values of Brillouin shift for cells^19–21,39^. When moving towards lamina propria, a sharp increase in Brillouin shift occurred: this region of the bladder was highly heterogeneous in ν_B_, with values distributed from 8 to 10 GHz, a higher range than the urothelium and that is consistent with other ECM values previously reported^26^. Finally, the bladder's muscle layer was characterized by a Brillouin shift ranging from 7.5 to 9.5 GHz. Notably, a gradient within the urothelium and muscle regions is evident in areas near the ECM. This phenomenon could be attributed to tissue layers not being sharply delineated and occasionally exhibiting mixtures of different components. Additionally, in the case of the muscle region, both perpendicular and parallel muscle bundles are present, which may give rise to different *ν*_*B*_ values.

### Temporal evolution of the mechanical properties of the healthy bladder wall

**Figure 3:**
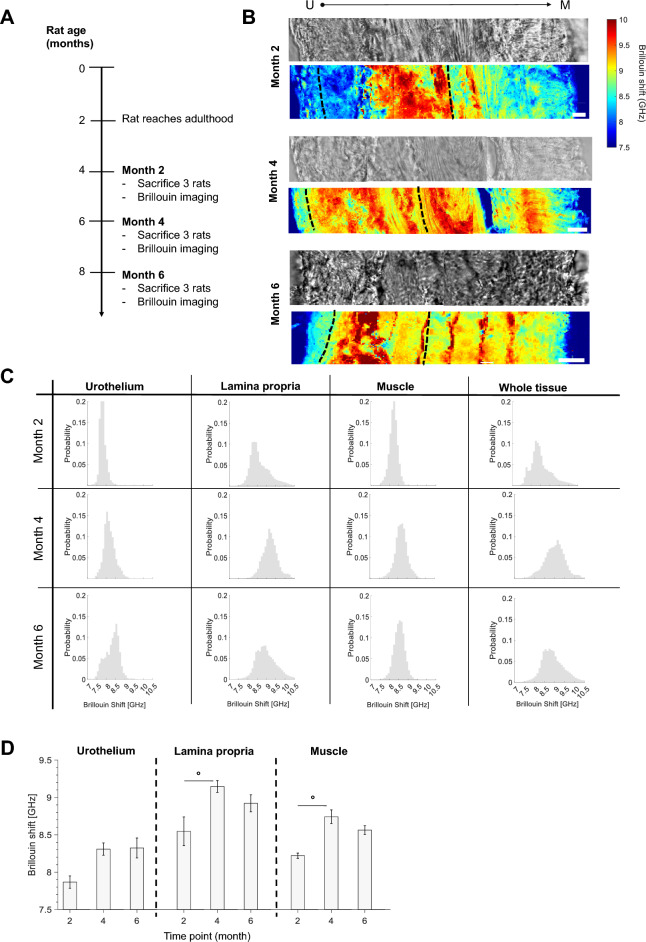
Brillouin imaging of healthy bladder walls at different ages of the adult rat. (**A**) Schematic representation of the experiment. Rats were sacrificed at different time points. (**B**) Representative healthy bladder DIC image and Brillouin shift maps of the urothelium, lamina propria, and muscle layer belonging to rats sacrificed at month 2, 4 and 6 (*i.e.*, 4, 6 or 8 months old rats, respectively). Scale bars = 20 µm. Different layer boundaries are indicated by dotted black lines. (**C**) Brillouin shifts distributions across different layers and at different time points. (**D**) Brillouin shifts histograms of medians of aging rats, divided by tissue layer. N = 3 rats per time point, each tissue layer of each single rat was characterized by over 4 Brillouin maps of at least 150 × 40 μm^2^. Brillouin shift of Lamina propria (p = 0.021) and Muscle layer (p = 0.026) significantly increased at month 4 with respect to month 2. (Mean ± SEM. ° *p* < 0.05, Kruskall-Wallis test followed by Dunn’s multiple-comparison test).

We characterized the impact of aging on bladder mechanical properties by imaging bladder tissue layers belonging to rats sacrificed at different ages (i.e., from 4 months old, which equals month 2 in our studied timeline, to 8 months old, which corresponds to month 6) (Fig. 3A). Representative DIC images and Brillouin shift images of rats sacrificed at different time points are shown in Fig. 3B. Brillouin shift distributions ranged from 7.5 GHz to 10.5 GHz and were characterized by several peaks whose location changed according to the tissue layer (Fig. 3C). When grouping together the medians of different tissue layers belonging to different samples (Fig. 3D), we found that Brillouin shifts of both lamina propria and muscle layers significantly increased from month 2 to 4, while no difference was seen at month 6. These data suggested that physiological aging in the adult life of the animals resulted in slight changes of lamina propria and muscle mechanical properties towards higher Brillouin shift values. The urothelium layer did not seem to change significantly between the studied time points.

### X-ray irradiation causing actinic cystitis results in a decrease of tissue Brillouin shift and lowers the Brillouin shift heterogeneity with respect to healthy animals

**Figure 4:**
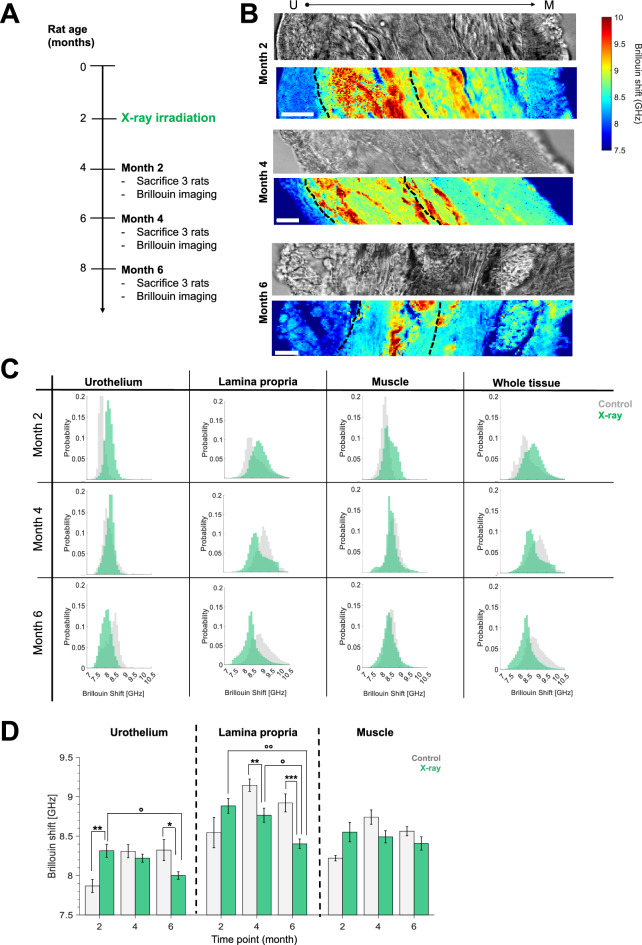
Brillouin imaging of murine bladder walls in a model of actinic cystitis (X-ray radiation) at different times post-irradiation. (**A**) Schematic representation of the experiment: 2 months old rats were exposed to X-rays and then sacrificed at different timepoints. Each timepoint matched the healthy counterpart reported in Fig. 3. (**B**) Representative fibrotic bladder DIC image and Brillouin shift map of the whole bladder wall (urothelium – lamina propria – muscle) from tissues belonging to rats sacrificed 2, 4, or 6 months after the irradiation. Scale bars = 20 µm. Different layer boundaries are indicated by dotted black lines. (**C**) Kinetics of Brillouin shift distributions from X-ray irradiated bladders at different time points (green) and comparison to healthy animals of the same age (grey). N = 1–3 rats per time point and condition, each tissue layer was characterized by over 4 Brillouin maps of at least 150 × 40 μm^2^. (**D**) Brillouin shifts medians of irradiated rats (green) compared to control rats (grey). When compared with their age-matched controls (here shown as * marks), the Brillouin shift of month 2 U was significantly higher than its control (p = 0.0095), while at month 6 it was lower (p = 0.045); L was lower both at month 4 (p = 0.007) and 6 (p = 0.0003) after X-rays. When comparing the effects of radiation on different stages of fibrosis (here shown as ° marks), U showed a significant (p = 0.011) decrease from month 2 to 6, while L both from month 2 to 6 (p = 0.0014) and from month 4 to 6 (p = 0.018). No significant changes in the muscle layer have been observed. (Mean ± SEM. For comparing fibrotic with their age-matched healthy counterpart, we used Mann–Whitney U-test: *, **, ***: *p* < 0.05, *p* < 0.01, *p* < 0.001. For comparing the stages of fibrosis between different timepoints, we used Kruskall-Wallis test followed by Dunn’s multiple-comparison test: °, °°: *p* < 0.05, *p* < 0.01.).

To model actinic cystitis onset, we here established a preclinical model in which 2-month-old rats were X-ray irradiated with a therapeutic dose and then sacrificed at 2, 4, and 6 months post-irradiation (Fig. 4A). Aiming to study early changes and progression of fibrotic processes, we started mechanical tests on the bladder at month 2. This decision considered that there is an inflammatory phase preceding fibrotic deposition of the matrix, which has been reported to alter bladder mechanics in the actinic cystitis model at 3 months after treatment^13^. We characterized the mechanical properties of irradiated bladders by Brillouin imaging (Fig. 4B) and plotted Brillouin shift distributions (Fig. 4C), and the medians of different layers belonging to different samples (Fig. 4D).

While the intrinsic mechanical heterogeneity within the bladder wall (differences in Brillouin shift from urothelium to lamina propria and muscle) was maintained (Fig. 4B,C), fibrotic remodeling of the bladder decreased Brillouin shift values for both urothelium and lamina propria; the muscle layer seemed not to be affected at any time point (Fig. 4C).

At month 2 the urothelium shift was significantly higher than untreated animals (Fig. 4C,D); also lamina propria displayed a higher shift than its age-matched control, although not significant.

This situation changed drastically at month 4 and 6, time points at which we found an overall decrease of Brillouin shift median values and distributions in comparison to untreated animals of the same age, in particular for lamina propria (Fig. 4C). The shape of the distributions of fibrotic bladder walls differed from the matching control ones (Fig. 4C): they were much less broad and tended to be bi- or single-modal instead of multimodal, thus showing a decrease in the tissue mechanical heterogeneity. Furthermore, lamina propria *ν*_*B*_ differences were significant not only compared to untreated tissues, but even when comparing the different treated time points between themselves: the longer the time after irradiation, the lower the Brillouin shift (Fig. 4D). Also, the urothelium’s Brillouin shift was significantly lower with respect to the control at month 6.

### Brillouin imaging and micro-indentation experiments provide complementary information on bladder wall mechanics

**Figure 5:**
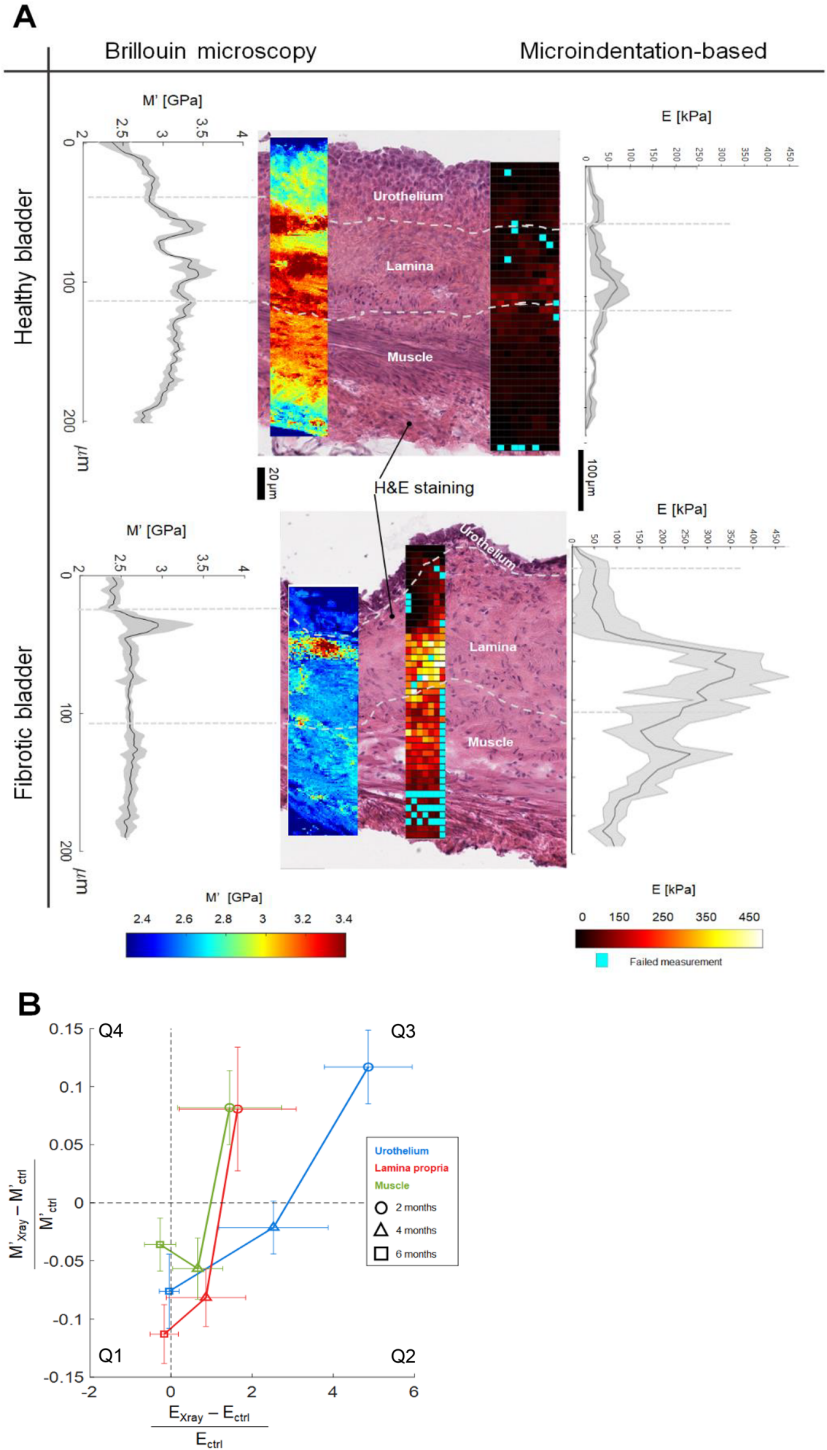
Comparison between Brillouin imaging and AFM-based microindentation experiments performed on the same bladder samples. (**A**) Brillouin Microscopy (left column) and microindentation-based (right column) imaging of murine bladder walls belonging to healthy (upper panel) and 4 months after X-ray (lower panel) rats**.** Investigation of mechanical properties of adjacent slides belonging to the same bladder, whose sample was prepared following the same protocol. We also report H&E staining sections of the same tissues performed after biomechanical acquisitions. The gradient curve of each map is shown alongside, depicting the mean ± SD across the section width. Respective color bars for each map are shown at the bottom. (**B**) Fold change ratio of Longitudinal and Young Modulus over time in the different layers. Q1-4: Quadrant number. Young’s modulus values here plotted have been adapted from this previously published study^14^ .

In an attempt to validate our results with the actual gold-standard technique of biomechanics, we compared Brillouin data to previously published microindentation data (sensitive to Young’s modulus E)^14^ (Fig. 5). By using the same sample preparation protocol and adjacent tissue slides from the same bladder organ, we here provided a robust experimental setup for comparing the information from the two different techniques, thus avoiding sample preparation artifacts introduced by different sample preparation protocols that could eventually affect mechanical properties measured by the two techniques. It is worth noting that Brillouin and indentation-based maps have different spatial resolutions. The resolution of the Brillouin microscope is determined by the employed high-numerical-aperture objective, the wavelength of the laser used, and the wavelength of the GHz acoustic waves in the tissues, which is below 1 μm; therefore, its three-dimensional resolution is diffraction-limited^29,40^ and, as discussed above, is 800 nm on the xy plane and ≈500 nm on the z-axis. In nanoindentation experiments, the spatial resolution is set by the contact radius, which, in the case of large spherical tips, is of the order of a few μm.

In order to provide a more meaningful comparison between the two techniques, we here transformed the Brillouin shift into Longitudinal Modulus by taking into account tissue densities and refractive indices of the different bladder tissue layers (see Methods).

When checking intrinsic mechanical variations within the healthy bladder wall, both Longitudinal Modulus (M’) and Young’s Modulus (E), measured by Brillouin microscopy and nanoindentation techniques^14^ , respectively, showed the same gradient (Fig. 5A): their values were both low in the urothelium, then increased in the lamina propria and decreased over the muscle layer, with their values orders of magnitude apart. The same behavior was observed when analyzing intrinsic variations within the fibrotic bladder wall in terms of M’ and E from urothelium to lamina propria and muscle layer. The overall agreement between M’ and E in healthy and fibrotic bladder samples was also shown in the log–log plot (Figure S3A) through a log–log linear fit: *log(M’/[Pa])* = a*log (E/[Pa]) + ***b***, where the fitting parameters were a = 0.071 and b = 9.08, similar to other values found in literature^30–32,39^.Interestingly, in this plot some points deviated from the expected correlation: in order to compare the said mechanical moduli values from healthy vs irradiated bladders, we produced a correlation plot showing the relative fold changes ΔE_rel_ = (E_Xray_-E_ctrl_)/E_ctrl_ and ΔM’_rel_ = (M’_Xray_-M’_ctrl_)/M’_ctrl_ of median values of both moduli (Fig. 5B), calculated from E and M’ values at different time points and layers (shown in Figure S3B). The quadrant in which the relative fold change is located depends on the sign of the numerator. If ΔE_rel_ and ΔM’_rel_ correlate, i.e. M’_X-ray_ > M’_ctrl_ and E_X-ray_ > E_ctrl_ or, viceversa, M’_X-ray_ < M’_ctrl_ and E_X-ray_ < E_ctrl_, their values are in the first and third quadrant (Q1 and Q3), depending whether there is a mutual tendency to be increased upon irradiation, or to decrease, respectively; otherwise, if they anticorrelate, i.e. M’_X-ray_ > M’_ctrl_ and E_X-ray_ < E_ctrl_, their values are plotted in the second or fourth quadrant (Q2 and Q4), respectively.

The comparison of the mechanical moduli from healthy to irradiated bladders revealed that at month 2 both E_X-Ray_ and M’_X-Ray_ were significantly higher than the controls in all the layers (values in the third quadrant and Figure S3B): E_X-ray_ was between two and five-fold higher than E_ctrl_, while M’_X-ray_ was between 0.1 and 0.12 fold times higher than M’_ctrl_: this is consistent with reported positive correlations between the two moduli in literature^30–32,39^. The scenario changed at month 4, where there was a tendency for E to increase in irradiated tissues while M’ decreased with respect to their controls (values in the second quadrant). This inversion of trends between E and M’ was interesting, yet not always statistically significant, because of the large error bars of our data (Figure S3). At month 6, E_X-Ray_ was unaltered with respect to controls, while M’_X-Ray_ was significantly and consistently lower than the untreated samples.

The same tendency towards a possible decoupling of the trends between E and M’ was observed in the gradient of the bladders maps (Fig. 5A) and in the distributions across the whole bladder tissue, showing that X-ray irradiation shifted E to higher values compared to the healthy bladder (Fig. 2 of reference^14^), while M’ shifted towards lower values (Fig. 4C).

## Discussion

Recent studies have revealed that changes in cell and tissue mechanical properties are hallmarks of many diseases^41^: the bladder is an excellent example of an organ in which alterations of mechanical properties critically impact its functions of urine storage and micturition, as they have to be tightly regulated on the urine volume. In this study, we exploited Brillouin imaging to characterize the mechanical properties of murine bladder tissues in healthy and fibrotic conditions. Analysis of tissue Brillouin spectra allows for a complete viscoelastic characterization by separating the elastic and viscous contributions.

Here, we reported: *i)* the intrinsic mechanical heterogeneity of the bladder wall at the microscale, dissecting the contribution of the different tissue layers; *ii*) the physiological aging effect on bladder mechanics; *iii*) alteration of bladder Brillouin shift on X-ray irradiated bladders in terms of a long term decrease in tissue Longitudinal Modulus M’; and *iv*) relation between median Longitudinal Modulus M’ and median Young’s Modulus E in healthy and X-ray irradiated murine bladders.

The healthy bladder wall was characterized by a gradient of Brillouin shift (Figs. 2 and 3), which revealed high mechanical heterogeneity of this tissue on its cross-section, from the urothelium to the lamina propria and muscle layer. Brillouin shift distributions were broad, ranging from 7.5 GHz to 10.5 GHz, and characterized by several peaks whose location changed according to the physiological layer. Such broad differences in mechanical properties between adjacent tissue layers of the bladder can be ascribed to their diverse biochemical scenario: cells in the urothelium are composed mainly of water, while the lamina propria is an interlaced mesh of water, proteoglycans, and fibrous proteins (primarily collagen I and III) and the outer layer of the bladder is formed by muscle bundles and fibers^37^. Healthy rat bladders at different ages showed higher values of Brillouin shift for lamina propria and muscle layers from month 2 to month 4 (Fig. 3C). These results go in line with what has been recently published regarding rat bladder static mechanics^14^, where Young’s Modulus (E) was recovered by microindentation based techniques from the very same rat bladders studied by Brillouin microscopy in this present paper. Intrinsic heterogeneity of the bladder wall was also reported (increasing gradient of E from urothelium to lamina propria and decrease over the muscle layer, as shown in Fig. 5A), as well as a general increase of E along with physiological aging of the animal.

We then characterized fibrotic bladders at different post-irradiation times (Fig. 4). Fibrosis was induced by X-ray exposure to mimic actinic cystitis, a pathological condition typical of cancer survivors, caused as a collateral effect of pelvic X-ray radiotherapy. Fibrosis is well known to be caused by the accumulation of extracellular matrix proteins, in particular of collagen^42^; however, its impact on bladder mechanical properties is still poorly described with high spatial resolution, and only very recently the contribution of the different anatomical components to the overall mechanical alteration in disease has been addressed by exploiting different mechanobiology techniques both at the macro^13^ and the microscale^14^. We here characterized the impact of fibrosis at different times after irradiation on Brillouin shift (Fig. 4). The fibrotic condition was qualitatively confirmed by H&E staining, while Brillouin microscopy could quantify differences between the investigated timepoints, particularly for lamina propria and urothelium. At month 2, the urothelium was the tissue component that mostly responded to X-ray irradiation, as its *ν*_*B*_ was significantly higher than the control, consistent with the cell membrane and DNA damage characteristic of cells that undergo X-ray irradiation^10^, shown to increase also Young’s modulus^43,44^. Lamina propria Brillouin shift was higher than the control, yet not significantly. The scenario changed at month 4 and more significantly at month 6: here, we measured a clear trend towards lower Brillouin shifts when comparing X-ray *ν*_*B*_ with control ones. Lamina propria Brillouin shift significantly decreased with respect to its matching control and to month 2 treated bladders. Moreover, lamina propria Brillouin shift distribution shapes changed dramatically, passing from a broad and multimodal distribution in healthy bladders towards a sharp and mono-modal distribution at later time points after X-ray irradiation. A similar behavior was found in the urothelium layer, whose Brillouin shift was significantly lower at month 6 with respect to its control and to month 2 treatment bladders.

We compared our results with those obtained by microindentation (the gold-standard technique of biomechanics) experiments, already published, performed on the very same rat bladders^14^. This was possible due to the use of a sample preparation protocol that allows for investigation both by spectroscopy and by microindentation techniques. For comparing with E, we transformed *ν*_*B*_ in M’ (real part of Longitudinal Modulus, see Methods) by including tissue densities and refractive indices of the different bladder tissue layers.

In healthy samples (Fig. 5A), M’ and E showed a very similar gradient, proving that the healthy murine bladder wall was characterized by a gradient of both elasticity E (as indicated by AFM-based experiments) and M’ (as indicated by Brillouin imaging). This is in good agreement with already established data on the correlation between M’ and E in cells^20,39^ and tissues^30–32,45–48^. In fibrotic samples, the intrinsic mechanical gradient of the bladder wall was maintained (Fig. 5A, Figure S3A); furthermore, we observed a decoupling between E and M’ trends. To quantify the agreement of E and M’ between fibrosis and control, we calculated the fold change ratio for Young Modulus (*i.e. (*E_X-ray_—E_ctrl_)/E_ctrl_ ) and Longitudinal Modulus (*i.e. (*M’_X-ray_ – M’_ctrl_)/M’_ctrl_) (Fig. 5B, based on data of Figure S3B). Here, the two moduli tended to be in good agreement at month 2, while at month 4 there was a tendency for M’ to decrease while E tended to increase upon X-ray irradiation. At month 6, E_X-Ray_ was unaltered with respect to controls, while M’_X-Ray_ was significantly lower than the untreated samples. It is important to note that these changes were not always statistically significant, as our data were characterized by large error bars (Figure S3), mainly due to animal-to-animal variability and to sample heterogeneity of the fresh-frozen tissue samples, which were not fixed or subjected to any additional treatments. These complex tissues displayed heterogeneous distributions in their physiological composition (presence of cells, muscles, nerves, blood vessels, ECM) and therefore in their mechanical moduli, particularly in microindentation data, where E spanned across multiple orders of magnitude (Fig. 2 of reference^14^). Nevertheless, the same trend observed in the mean values was corroborated by the distributions of the values (Fig. 2 of reference^14^ and Fig. 4C) and in the gradient of the bladders maps (Fig. 5A).

Thus, our findings suggest the possibility of a decoupling between E and M’ trends at different time points, that indeed deviated from the expected log–log linear correlation^30–32,39^, as shown in Figure S3A. In literature, a positive correlation between E and M’ in tissues has been mainly found at a specific time point^30–32,44,46–48^; here, we follow the evolution in time of bladder mechanical properties in untreated and irradiated animals, a set-up that includes a layer of complexity that was not reported in previous studies.

This tendency of decreased M’ towards later fibrotic stages could seem in principle counterintuitive: recent biomechanical studies, performed at the microscopic level with microindentation^14^ or macroscopically with a mechanical stretcher^13^, revealed indeed that X-ray irradiation of the bladder should result in a higher Young Modulus of the whole tissue^14^ and in a lower distensibility of the lamina propria^13^.

We here propose that this decoupling between E and M’ trends might be explained in the approach used to recover M or E in spectroscopy- and microindentation-based techniques. The Young’s modulus E measured by nanoindentation techniques like AFM describes the elastic response of the sample to normal compressive stresses in the low-frequency regime (nearly static compression in our case, or up to 300 Hz in microrheological measurements), where the volume is kept constant. On the contrary, Brillouin light scattering measures the longitudinal elastic modulus M, which describes the elastic response of the material to oscillating high-frequency (GHz) longitudinal stresses (the traveling acoustic waves), where the system's volume (or density) is changed when applying the stress. Therefore, the two techniques sense material elasticity in very different frequency ranges: microindentation in the 1–100 Hz regime, Brillouin in the GHz regime, which justifies the large difference in the values of the two elastic moduli seen in Fig. 5A,.i.e. kPa for E and GPa for M.

In viscoelastic materials, the response to a mechanical stimulus depends on the frequency at which it is applied^2^. Importantly, cells, tissues and ECMs have been proved to be viscoelastic materials and to exhibit complex mechanical properties^16,49^. In a viscoelastic material, both E and M increase almost stepwise when the frequency of the probe ***ν***_*0*_ becomes comparable to the inverse of sample relaxation time ***τ***_*relax*_. Without entering in the details (the interested readers can refer to standard textbook on the matter^50^) it is sufficient to mention that in condensed matter ***τ***_*relax*_ falls in the ps-ns time scale, depending on the aggregation state (liquid, glasses, …) of the probed region. While to a good extent E measured in AFM-based microindentation experiments is measured at ***ν*** <  < 1/***τ***_*relax*_, M’ is often strongly frequency dependent. Therefore, a change in ***ν***_*B*_ in a viscoelastic transition is seen when ***ν***_0_*τ_*relax*_ ≈ 1, which can happen if the relaxation time ***τ***_*relax*_ is in the 0.1 ns range^49,51^. Molecular dynamics simulations have proved that collagen relaxation time at high frequencies falls indeed in this range^52^. X-ray irradiation is known to induce crosslinking in the collagen structure^53^, thereby increasing its relaxation time^53–55^ . In this work, we irradiated rat bladders and monitored irradiation effect on mechanical properties during time: at month 2 we observed higher Brillouin shifts compared to controls, which may possibly be related to collagen crosslinking and its subsequently higher relaxation time. As time progressed, the bladders might have undergone unidentified physiological mechanisms that could have impacted on their structure in various ways, such as reducing collagen relaxation time or altering the tissue viscoelastic or chemical-physical parameters, ultimately resulting in a decrease of M’. Consequently, our data may suggest that the biophysical modifications caused by X-rays over time have a broad impact on bladder tissues’ relaxation time at high frequencies, passing from a condition where ***τ***_*relax*_ ≈ 100 ps in healthy samples, towards another one in which ***τ***_*relax*_ > 100 ps right after X-ray irradiation and then, possibly, ***τ***_*relax*_ < 100 ps at month 4 and 6 in fibrotic samples.

Changes in ν_B_ have been established to be directly dependent on actual variations in M’ in biomatter^20,45,48,56^; nonetheless, under certain circumstances the Brillouin shift may be influenced also by various physical parameters of the sample. For example, the hydration level can impact the Brillouin shift^57^; in our samples, however, there should be minimal variation in water content from healthy to fibrotic conditions, and even if alterations in water content were to exert a significant influence on ν_B_, we believe that it would similarly impact E measurements on the same samples^57–61^. Another important factor influencing ν_B_ is *ρ/n*^2^ factor (where *ρ* = density, *n* = refractive index; see Methods): in our study, we assumed that this ratio remained constant across the bladder section and from wild-type to fibrotic conditions, an reasonable approximation for biological samples and well-validated in literature^38,46^, corroborated by the fact that fibrotic process in the bladder so far has never been described as leading to changes in *n* or *ρ.* However, we cannot exclude that a change in *ρ* might possibly occur in the presence of X-ray-induced cross-linking^62^. Moreover, *ρ/n*^*2*^ significantly changes in the presence of lipids, where a higher ν_B_ corresponds to a lower M’^63^: however, bladder fibrosis is mainly characterized by the accumulation of collagen in the ECM^10^, while changes in lipid concentrations from healthy to actinic cystitis conditions in bladders have not been so far documented, to the best of our knowledge. Thus, further studies would be necessary to establish the influence of water content, density, and refractive index on bladder fibrosis.

Fibrotic processes are characterized by increased collagen deposition, which in the bladder results in increased micturition frequency and reduced urine volume^14^. It has been previously published that the kinetic behavior of E in the lamina propria of fibrotic bladders was associated with increased collagen deposition: at months 2 and 4, where both E and collagen deposition were higher than in control bladders, while at month 6 they both were not significantly changed, due to physiological aging of both control and irradiated animals^14^ (see Fig. 2 of reference^14^). Instead, *ν*_*B*_ trend did not follow the same trend as collagen deposition in the lamina propria: *ν*_*B*_ alteration was even more dramatic at month 6, where no differences in collagen deposition between irradiated and control animals were observed. Thus, our results suggested that *ν*_*B*_ and therefore M’ behavior is not exclusively dependent on different collagen deposition in the bladder lamina propria. The cause-effect relationship between tissue mechanics and collagen deposition is indeed still not clear, as some research in different fibrotic tissues (lungs and liver) reported higher values of elastic moduli as a simple consequence of increased collagen deposition^64–67^, while other studies in fibrotic liver suggested that elasticity and collagen levels are not linearly correlated^68^ and that, in turn, the increase in elasticity precedes the deposition of collagen^47^. Furthermore, augmented collagen deposition in cornea tissues has been proved to cause a rapid increase of Brillouin shift^30,69^, while our data (following the kinetics of already established inflammation) suggested that the collagen deposition in the bladder upon chronic inflammation is not related to Brillouin shift. A recent research^70^ performed with quantitative micro-elastography (which microscopically maps tissue elasticity) on two different murine models of chronic liver fibrosis clearly showed that fibrotic and cirrhotic regions (correlated with collagen deposition) had increased tissue elasticity compared to surrounding areas; interestingly, these data are highly consistent with our reported M’ data.

A major limitation of the present research is the treatment of *ρ/n*^*2*^ ratio and hydration level as constant factors in the calculation of M’ irrespective of whether they were applied to control or fibrotic samples, but these might be of difficult measurement in physiological bladder tissues. Another possible limitation of our study is that bladder tissues were snap–frozen before mechanical measurements. Although samples were considered fresh/frozen as no additional treatment was performed, the freezing/unfreezing protocol could still include artifacts on the sample. However, working with frozen tissue presents several advantages such as compatibility with histological examination and sample transport and storage. When measuring fresh/frozen tissues, there is a limitation on the time that we can spend performing experiments on them. In addition, bladder tissue experiments using the different techniques here reported and previously published^14^ were performed in different cities. Given also the heterogeneity of the tissue, which differs mechanically even in the same macro-regions of the tissue, we believe that the best system is measuring consecutive tissue slides of the same bladder organ in many different regions, as here performed. Aiming to minimize this spatial variation, we investigated consecutive slides by Brillouin imaging and Young’s modulus investigation^14^, in order to provide a good comparison between Longitudinal and Young’s moduli by measuring the same macroscopical tissue region.

## Conclusions

In this study, we characterized the mechanical changes of the murine bladder in the presence of actinic cystitis, a pathological condition well known to dramatically affect bladder function and cancer survivors' quality of life after pelvic X-ray radiotherapy. Here, we demonstrated that X-rays exposure, leading to fibrosis, has a broad impact on Longitudinal Modulus which Brillouin Microscopy was able to quantify with high sensitivity, as well as to discriminate between different time points post X-ray irradiation resulting in different stages of fibrotic mechanical alteration. Such accuracy is missing in standard imaging techniques such as DIC or H&E staining, indicating that fibrosis consists of a progressive condition, from a mild stage at lower times post-irradiation to a severe one at longer times, and that it may induce a leveling in mechanical properties heterogeneity, as already proposed in recent studies^8,13,71^.

By providing a direct comparison between the gold standard technique in nanomechanics (microindentation-based measurements) and Brillouin microscopy, we here aimed to improve the translatability of such mechanical tests to the clinics as a potential diagnostic and prognostic tool while detecting complex viscoelastic properties of biomatter at the microscale^30,49^. Our data may suggest that the biophysical modifications caused by X-rays over time determine a non-trivial correlation between E and M’. This work could pave the way to exploiting Brillouin microscopy to monitor the mechanical changes of bladder tissues and to use this mechanical marker for screening and diagnostic purposes.

Future developments of this work would pave the way for a deep understanding of the mechanical properties of the bladder, fundamental for artificial bladder implementation of patients that underwent radical cystectomy due to actinic cystitis or bladder tumor, that could mimic bladder physiological properties closely and produce functional artificial bladders that will notably improve the quality of life of patients. These results could also be crucial for the early detection of collateral effects of pelvic X-ray radiotherapy.

## Materials and methods

### Actinic cystitis model establishment-radiotherapy and sample preparation for mechanical analysis

All procedures and studies involving rats were approved by the Institutional Animal Care and Use Committee of San Raffaele Scientific Institute and performed according to the prescribed guidelines (IACUC, approval number 942), and performed according to ARRIVE guidelines. Adult (9–10 weeks old) female Fischer rats were from Charles River Laboratories, Italy. Radiotherapy was performed as recently reported^14^.

Sample preparation for tissue mechanics assessment by microindentation and Brillouin imaging was performed as reported elsewhere^14^. Briefly, ultrasound imaging^72^ of the rat bladder was performed to determine the physiological volume resulting in a physiologically stretched state of the bladder. Animals were then euthanized by CO_2_, bladders were injected with OCT in vivo before explanation and frozen in the cryoprotectant OCT. 50 µm thick cryosections of the bladder were prepared and attached to glass slides. Consecutive slides were prepared for Brillouin imaging and for Young’s Modulus investigation (Young’s Modulus results reported in detail in this previously published study^14^), to provide a good comparison between Longitudinal and Young’s modulus by measuring the same macroscopical tissue region. Before measurements, the cryoprotectant was removed by two PBS washes for 10 min, and the tissues were always kept in liquid. A glass coverslip was attached to the top of the tissue section and sealed with nail polish. Haematoxylin Eosin (HE) staining was performed on consecutive tissue slides as well as in the measured slides after Brillouin imaging was performed as described previously^14^.

### Brillouin imaging

Working in a backscattering configuration as in our Brillouin Microscope, the Brillouin spectrum (Fig. 1B) is characterized by the Brillouin frequency shift ν_B_ with respect to the laser radiation and by the Brillouin Full Width at Half Maximum Γ_B_ , both related to M’ and M’’ via these equations:

1 $${\nu }_{B}=\sqrt{\frac{{M}{^\prime}}{\rho }}*\frac{2n}{\lambda }$$

2 $${\Gamma }_{B}=\frac{{M}^{{\prime}{\prime}}}{\rho {\nu }_{B}}{\left(\frac{2n}{\lambda }\right)}^{2}$$

where *n* is the sample index of refraction, *ρ* the sample density, λ the laser wavelength (here, λ = 532 nm). Changes in ν_B_ have been proved to be directly dependent on actual changes in M’ in biomatter^20,45,48,56^ since $n/\sqrt{\rho }$ ≅ constant in biological systems.

Our custom-built Confocal Brillouin Microscope (Figure S1A) consists of an inverted microscope (Olympus IX-73) coupled to a single virtually imaged phased array (VIPA)-based spectrometer^35^ through single-mode optical fibers. The 3D mechanical properties of the sample are recovered in confocal laser scanning mode thanks to a pair of galvanometric mirrors (THORLABS) and a piezo stage (MadCity Labs). The laser source is a continuous-wave single-mode laser at a 532 nm wavelength (COHERENT VERDI). The laser passes through a polarizing beam splitter, the galvo mirrors, and a quarter wavelength plate and then is focused on the sample plane via a 60 × objective (Olympus, NA = 1.40) to have high-resolution Brillouin maps of biological samples, working in backscattering configuration to ensure minimum spectral broadening^73^. The retrieved Brillouin and Rayleigh signals, scattered from a specific point of the sample, are then coupled to a single-mode fiber through a 20 × objective. The core of this optical fiber (diameter = 3.2 micron) acts as a pinhole and allows for confocal sectioning of the sample: thus, with λ = 532 nm and NA = 1.4, the spatial resolution of Brillouin maps is comparable to confocal ones (~ 400 nm on *xy* plane, ~ 500 nm on *z* ^36^*).*To suppress the background signal, *i.e.* the Rayleigh component, a glass prism is used to realize a wavefront splitting interferential filter^74^. Despite the filtering, the Rayleigh line is hardly ever totally suppressed in highly scattering materials such as cells. The filtered signal is then spatially dispersed by the VIPA^35^ (LightMachinery) in the spectrometer, having a Free Spectral Range (FSR) of 30 GHz, whose Gaussian envelope is seen in all the Brillouin spectra acquired (Fig. 1B).

Aside from the Brillouin laser-scanning module, a standard brightfield unit is located in the Microscope: a lamp and a camera for widefield transmission images in brightfield or Differential Interference Contrast (DIC) mode to retrieve morphological information of the sample.

The setup is controlled in MATLAB via a custom-built graphical user interface. During data acquisition, the stage longitudinal step size on the sample was 800 nm, the acquisition time was 100/150 ms per point, and the optical power delivered to the specimen was 25 mW.

We adjusted the VIPA angle to allow the transmission of a couple of adjacent orders of Rayleigh and Brillouin peaks: thus, our Brillouin spectra (Fig. 2A) are typically composed of 2 Rayleigh curves of adjacent dispersion orders, fixed at 0 and 30 GHz, and 4 Brillouin peaks (i.e., a Stokes and Anti-Stokes signal per Rayleigh order) that change in shape according to the local point (Fig. 2B). The advantage of keeping Rayleigh peaks in the spectrum is to use them as a reference to correct for laser drifts during time, thus avoiding frequent calibrations from standard materials that slow down the overall data acquisition, which is particularly relevant when using non-fixed tissues. After pixel-to-GHz conversion, obtained from distilled water calibration of the spectrometer, we fitted data with a sum of Pseudo-Voigt for Rayleigh components and of pure Lorentzians for Brillouin curves. The spectral precision of the microscope was 16 MHz at 80 ms and 20 mW power on the sample plane (Figure S1B), measured with water Brillouin shift’s distribution standard deviation^63^, while its signal-to-noise ratio was 17, calculated as the Brillouin water spectrum average intensity of the maximum divided by the standard deviation of the same point^63^.

Bad spectra pixels in the maps were isolated points (generally, less than 5% of the pixels composing a map) coming from saturated Rayleigh peaks. The missing values in these pixels have been recovered from the mean of the Brillouin shifts of 8 neighbors surrounding a saturated point.

No other manipulations have been applied to maps for visualization of data. All data analysis has been performed using custom-made MATLAB codes.

### Brillouin imaging for bladder tissues

For bladder tissue acquisitions on our Brillouin Microscope, we thawed the samples (treated as described in Section “Actinic cystitis model establishment-radiotherapy and sample preparation for mechanical analysis“) and removed OCT cryoprotectant with several washes in PBS. After adding some final drops of PBS, we added a 0.17 mm coverslip to allow high-numerical aperture objective imaging and sealed its borders with nail polish. In such a manner, the buffer never evaporated and kept the tissues fresh and hydrated during long acquisitions. Its presence throughout the acquisition was confirmed by its Brillouin spectrum, which is similar to water (Fig.  2B). We first chose the bladder portion to investigate through the DIC imaging module via a large field of view (i.e., a 10 × objective). Then, we switched to a high magnification, small field of view (60x, NA = 1.4) objective, also used for Brillouin acquisition. Bladder wall was evaluated in different regions per slice, whose location under the microscope was achieved by optical transmission microscopy in differential interference contrast (DIC) mode. By simultaneously imaging the tissue in DIC mode, the whole thickness of the bladder wall was evaluated, from edge to edge. Hematoxylin eosin stained consecutive slides (prepared as reported in 14 ) confirmed the different tissue layers appreciated in the DIC images and Brillouin maps (as shown in Fig. 2C).

Brillouin data were acquired all at the same depth, i.e., 5 microns from the PBS buffer surface. We always checked the presence of PBS on the surface of the tissues through its Brillouin spectrum, similar to water and very different from the sample, and then we entered for 5 microns, where we always had a good SNR.

### Brillouin shift-longitudinal modulus conversion

The Brillouin shift has been experimentally proved to be an optimal proxy for M’ in biomatter^20,45,48,56^: according to Eq. (1) below, $\rho /{n}^{2}$ is required for converting ν_B_ in M’. From literature values of the refractive index and density of urothelium, lamina propria and muscle in the bladder wall, we found:

- Urothelium: *n* = 1.44^75^, $\rho$ = 1.040 g/mL^76^; $\rho /{n}^{2}$ = 0.5015 g/mL
- Lamina propria: *n* = 1.41^77^, $\rho$ = 1.040 g/mL^76^; $\rho /{n}^{2}$ = 0.5231 g/mL
- Muscle: *n* = 1.39^78^, $\rho$ = 1.050 g/mL^76^; $\rho /{n}^{2}$ = 0.5435 g/mL

Since the variation of this values is less than 4%, we chose to take the mean value of $\rho /{n}^{2}$ = 0.52 g/mL for converting ν_B_ to M’ in Fig. 5.

### Indentation techniques

The Young’s modulus of bladder tissue specimens have been characterized using the Bioscope Catalyst AFM (Bruker) and the Chiaro nano-indenter (Optics11), in a previously published study here reported as reference^14^. Results from reference^14^ have been adapted and partially elaborated to calculate relative variations. We refer the reader to the previously published study for more information on Young’s modulus investigation; briefly, the tip radius used was 5–10 µm, with an indentation depth of 2–5 µm. Data was fit using the Hertz model^79,80^.

### Statistical methods

We acquired at least 4 sections per slice, at least 2 slices per rat bladder, n = 3 rats per time point and condition.

For every section, after the division in urothelium/lamina propria/muscle based on their Brillouin shift, we calculated the median value of each layer, and we repeated it for every condition., In the end, we averaged the values obtained and graphed their mean $\pm$ SEM in histograms.

All statistical analysis has been performed using custom-made MATLAB codes. We checked the normality of data with a Kolmogorov–Smirnov test; no data was found to be Gaussian. We used Kruskall-Wallis test followed by Dunn’s multiple-comparison test to compare control or fibrotic conditions between different time points. For comparing fibrotic with age-matched healthy counterparts, we used non-parametric Mann–Whitney U-test.

### Supplementary Information

Supplementary Figures.

## Data Availability

The datasets generated and/or analyzed during this study are available from the corresponding author on reasonable request.
