# Supplementary material for: Progressive alteration of murine bladder elasticity in actinic cystitis detected by Brillouin microscopy
Source: Sci Rep. 2024 Jan 4;14:484. doi: 10.1038/s41598-023-51006-2 (PMC10766652; doi:10.1038/s41598-023-51006-2)
Supplement: Supplementary file 1 — Supplementary Figures. [file 41598_2023_51006_MOESM1_ESM.pdf]

SUPPLEMENTARY INFORMATION for the manuscript "Progressive alteration of murine bladder elasticity in actinic cystitis detected by Brillouin Microscopy"

Laura Martinez-Vidal*#, Claudia Testi*#, Emanuele Pontecorvo, Filippo Pederzoli, Elisa Alchera, Irene Locatelli, Chiara Venegoni, Antonello Spinelli, Roberta Lucianò, Andrea Salonia, Alessandro Podestà, Giancarlo Ruocco#, Massimo Alfano#

#equal contribution

*correspondence

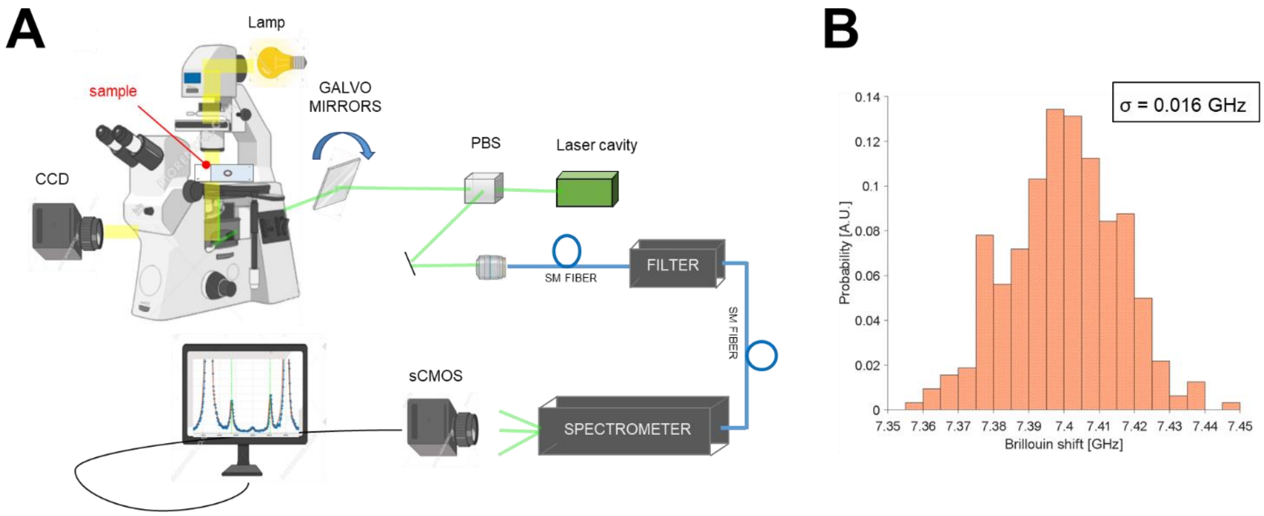

Figure S1: Experimental setup for Brillouin imaging. A) Our microscope consists of a standard brightfield unit (left, consisting of a lamp and a CCD camera) and a Brillouin unit (right) mounted on two different ports of an inverted microscope (Olympus IX-73). The sample is placed on a stage with a moving z-scan piezo (Mad City Labs). The radiation (CW laser, λ = 532 nm, COHERENT VERDI) passes through a polarizing beam splitter (PBS), galvo mirrors (THORLABS) and a quarter wavelength plate and then is focused on the sample plane via a 60x objective (Olympus, NA = 1.4). The signal scattered from a point of the sample is then deflected by the PBS and then coupled through a 20x objective to a single-mode fiber (diameter = 3.2 micron) that acts a pinhole, ensuring confocal resolution to our Brillouin maps. The Rayleigh signal is then interferometrically attenuated by a quartz prism. Finally, the Brillouin signal is spatially separated from Rayleigh through a single VIPA (FSR = 30 GHz) and then imaged on a sCMOS. **B)** Spectral resolution of our microscope, measured with water Brillouin shift's distribution standard deviation[60].

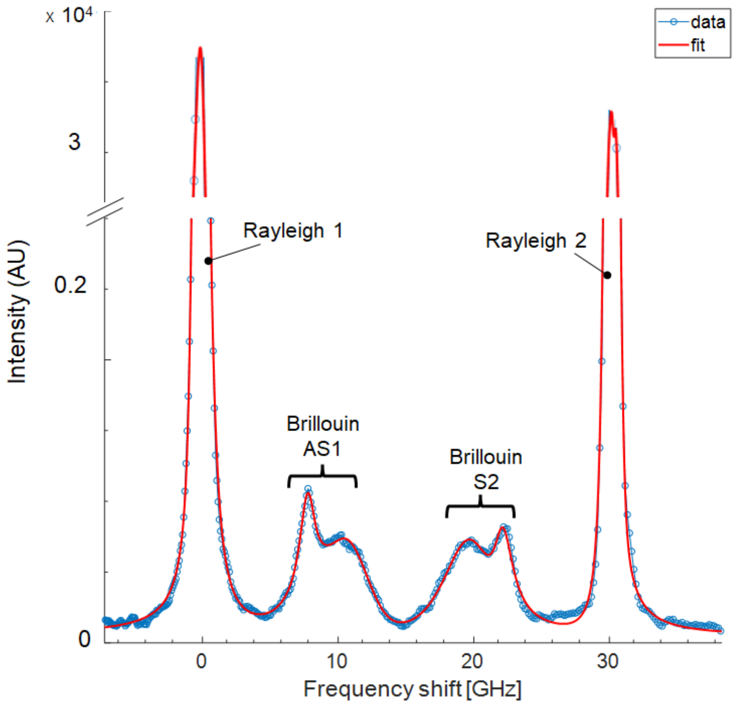

Figure S2: **Brillouin blended spectra of two materials.** In the rare cases where spatial changes of the sample occurred at a scale smaller than our resolution, we observed a mixture of signals similar to the one here shown. We detected indeed similar signals in cases where ECM layer contained few cells. Here, each Stokes and Anti-Stokes Brillouin curve is composed by 2 peaks, whose parameters from the fit (red lines) are: Brillouin 1: shift 7.73 GHz, FWHM 1.14 GHz (typical of cells); Brillouin 2: shift 10.50 GHz, FWHM 3.98 GHz (typical of wild-type ECM).

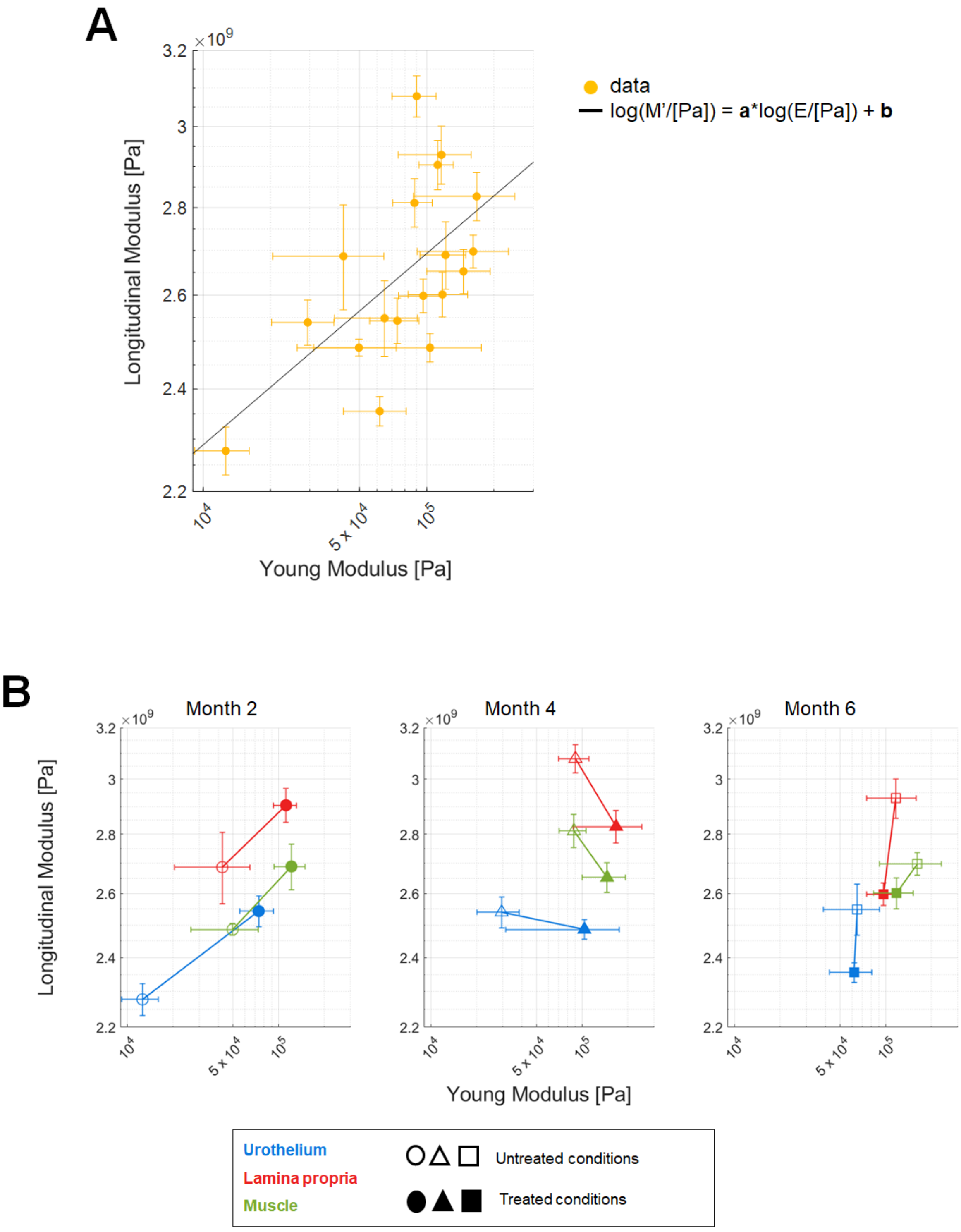

A
data
log(M'/[Pa]) = a*log(E/[Pa]) + b
Longitudinal Modulus [Pa]
Young Modulus [Pa]
B
Month 2
Month 4
Month 6
Urothelium
Lamina propria
Muscle
Untreated conditions
Treated conditions

		Month 2	Month 4	Month 6
$M'_{x\text{-}ray}$ *vs* M'_{ctrl}	U	**p=0.0095**	p=0.073	**p=0.045**
	L	p=0.059	**p=0.007**	**p=0.0003**
	M	p=0.065	p=0.079	p=0.160
$E_{x\text{-}ray}$ *vs* E_{ctrl}	U	**p=0.0047**	p=0.153	p=0.89
	L	**p=0.0151**	p=0.180	p=0.51
	M	**p=0.038**	p=0.113	p=0.390

Figure S3: Log-log correlation plots of E and M'.A) Log-log linear relationship between E and M' through the fit: log(M'/[Pa]) = a*log(E/[Pa]) + b: fitting values were found to be a = 0.071 and b = 9.08. **B)** Some points in A) deviated from the expected relationship: here, the data are categorized in different time intervals and layers, with unfilled data points representing untreated conditions and solid data points representing treated samples. We used these values to calculate ΔE and ΔM' shown in **Figure 5B**; Young's modulus values here plotted have been adapted from this previously published study[14]. At month 2, both M' and E exhibited higher values in treated conditions with respect to untreated conditions, as evidenced by the positively sloped lines. This trend, however, undergoes a shift at month 4, leading towards lower values for M' and higher values for E, indicated by negative sloped lines for all the layers. At month 6, E displayed almost no distinction between treated and untreated samples, while M' was consistently lower. In the table, we show the p-values between fibrotic and control conditions, calculated by Mann-Whitney U-test, where we highlighted in bold all the statistically significant values ($p<0.05$).
